# Prognostic significance of *CUX1* genomic deletion in myelodysplastic neoplasms

**DOI:** 10.1007/s00277-026-06936-y

**Published:** 2026-03-13

**Authors:** Mohamed M. Khamis, Aleksandar Babic, Aref Al-Kali, Omar Alkharabsheh

**Affiliations:** 1 Department of Medicine, Mercy Hospital St. Louis, St. Louis, MO USA; 2 Hematology/Oncology, Blood and Marrow and Cellular Therapy Program, SSM Health Saint Louis University Hospital, St. Louis, MO USA; 3 Division of Hematology, Mayo Clinic, Rochester, MN USA; 4 Division of Hematology/Oncology, University of Cincinnati College of Medicine, Cincinnati, OH USA

**Keywords:** Myelodysplastic neoplasms, MDS, CUX1, Copy-number loss, Prognosis, IPSS-M

## Abstract

**Background:**

Molecular profiling has transformed risk stratification in myelodysplastic neoplasms (MDS). However, the independent prognostic value of specific genomic alterations beyond comprehensive molecular scoring remains unclear. We investigated whether *CUX1* copy-number loss (haploinsufficiency) adds prognostic value and its association with other mutations. Given its correlation with chromosome 7 abnormality, we examined its independent significance within the current MDS molecular framework.

**Methods:**

A cohort of 501 MDS patients with available *CUX1* copy-number data (CNACS gene-level calls) and complete clinical follow-up was analyzed from cBioPortal. We assessed associations with clinical characteristics, co-occurring alterations, and survival. Cox proportional hazards models evaluated independence adjusting for IPSS-R and IPSS-M.

**Results:**

*CUX1* loss occurred in 129/501 patients (26%), nearly always with − 7/del(7q) (98%). Patients with *CUX1* loss had higher marrow blasts (median 7% vs. 4%, *P* < 0.001), IPSS-R scores (6.9 vs. 4.6), and IPSS-M scores (2.33 vs. 0.79). *CUX1* loss strongly associated with *EZH2* alterations (OR 223.8) and complex karyotype (60%). In univariable analysis, *CUX1* loss predicted inferior overall survival (OS; median 11.8 vs. 27.1 months; HR 2.39, 95%CI 1.85–3.08, *P* < 0.001) and leukemia-free survival (LFS; HR 2.30, 95%CI 1.78–2.98, *P* < 0.001). After IPSS-M adjustment, associations were non-significant (OS HR 1.27, *P* = 0.11; LFS HR 1.23, *P* = 0.15) with negligible incremental discrimination. Within the − 7/del(7q) subgroup, no survival difference was detected (OS *P* = 0.41; LFS *P* = 0.31).

**Conclusion:**

*CUX1* loss identifies high-risk MDS with − 7/del(7q) and *EZH2* co-alterations but provides no independent prognostic information beyond IPSS-M. Isolated *CUX1* deletions are rare. *CUX1* loss reflects − 7/del(7q) biology rather than independent prognostic significance.

**Supplementary Information:**

The online version contains supplementary material available at 10.1007/s00277-026-06936-y.

## Introduction

Myelodysplastic neoplasms (MDS) are clonal bone marrow disorders characterized by ineffective hematopoiesis, peripheral blood cytopenias, and variable risk of progression to acute myeloid leukemia (AML) [1]. Clinical outcomes range from indolent disease requiring minimal intervention to rapidly progressive leukemia requiring intensive therapy. This heterogeneity has driven the development of prognostic scoring systems, including the Revised International Prognostic Scoring System (IPSS-R) [2], and more recently, the Molecular International Prognostic Scoring System (IPSS-M) [3]. IPSS-M incorporates somatic mutations alongside cytogenetic and clinical variables.

Risk stratification has evolved substantially. The original IPSS (1997) categorized patients into four groups with median overall survival (OS) ranging from 0.4 to 5.7 years [4]. IPSS-R (2012) introduced a five-tiered scheme with OS ranging from 0.8 to 8.8 years and refined cytopenia/blast scoring [2]. IPSS-M (2022) integrates clinical variables, cytogenetics, and 31 recurrently mutated genes, yielding six risk categories.

Within IPSS-M, − 7 and del(7q) are encoded as distinct cytogenetic variables [3]. Polycomb Repressive Complex 2 (PRC2) component mutations (e.g., *EZH2*) receive gene-specific adverse weights [3]. *CUX1*, located at 7q22, is not separately encoded because its loss almost always occurs within broader − 7/del(7q) lesions [5, 6].

The 7q region harbors several genes implicated in myeloid neoplasia, including *CUX1* (Cut Like Homeobox 1), *EZH2*, *SAMD9*, and *SAMD9L* [7]. Contemporary genomic studies support a contiguous haploinsufficiency model [7–9]. Cumulative loss of multiple 7q tumor suppressors (*CUX1*, *EZH2*, *LUC7L2*, *KMT2C*/*MLL3*, among others) drives disease pathogenesis through impaired hematopoietic stem-cell function, defective DNA repair, and epigenetic dysregulation. A recent multinational analysis of 519 AML patients with chromosome 7 aberrations identified *KMT2C* as one of the most frequently mutated genes (16%), alongside *EZH2* (10%), with mutations often subclonal and enriched in del(7q) [10]. These findings underscore the importance of coordinated 7q gene dysregulation in myeloid transformation.

*CUX1* encodes a transcription factor with roles in cell proliferation, differentiation, and tumor suppression [11]. Several studies have demonstrated the prognostic relevance of *CUX1* due to its role in DNA repair and tumor suppression [12, 13]. *CUX1* loss leads to reduced gene expression, promoting tumorigenesis [11]. *CUX1* alterations occur across myeloid neoplasms, including AML, chronic myelomonocytic leukemia (CMML), and MDS [7, 12].

The prognostic significance of *CUX1* alterations in MDS has been inconsistent across studies: some reports demonstrate an association with inferior survival [12], while others have not found a significant independent prognostic effect [14]. These contradictory findings may reflect differences in the type of *CUX1* alteration examined (point mutations versus copy-number loss), variation in cohort characteristics, or incomplete adjustment for established prognostic factors.

Recent work from the Cleveland Clinic has highlighted distinct patterns associated with *CUX1* mutations versus deletions, with mutations primarily associated with high-risk MDS and deletions correlating with increased blast counts [12]. Co-alteration analyses have revealed associations with *TET2*, *U2AF1*, PRC2 complex members (*EZH2*, *EED*, *SUZ12*), and RAS family genes (*NRAS*, *KRAS*) [12].

In this study, we examined a large cohort of patients with MDS from cBioPortal [15]. We focused on *CUX1* copy-number loss, as determined by CNACS (Copy Number Alteration Calling System) gene-level analysis, to address the contradictory survival findings and assess the incremental prognostic value of *CUX1* loss beyond IPSS-M. We also characterized the co-alteration landscape and phenotypic associations of *CUX1* loss.

## Methods

### Study design and data source

We conducted a retrospective cohort study using the publicly available MDS dataset curated in cBioPortal. We extracted 3,466 unique patients and restricted survival analyses to those with available overall survival (OS) or leukemia-free survival (LFS) information. Patients with available CNACS gene-level calls for *CUX1* copy-number status comprised the primary analysis cohort (*n* = 501; 129 with loss, 372 without loss).

### Ethics and approvals

This study used only de-identified, publicly available data from cBioPortal. No additional ethics approval was required, as the original source studies had obtained appropriate ethics approvals and informed consent.

### Additional data integration

To address treatment and molecular characterization, we cross-referenced our cohort with the source IPSS-M repository (Bernard et al. [3]), matching 497 of 501 patients by anonymized identifiers. This provided treatment annotations (hypomethylating agents ^HMA^, allogeneic hematopoietic stem cell transplantation ^HSCT^, lenalidomide, intensive chemotherapy), *TP53* allelic state (monoallelic versus multi-hit, as defined by Bernard et al. [16]), and point mutation data for 130 recurrently mutated genes. The IPSS-M source repository is publicly available at: https://github.com/papaemmelab/IPSSMstudy.

### Variables and exposures

*CUX1* alterations were derived from CNACS gene-level copy-number calls: cux1_loss (copy-number deletion), cux1_upd (uniparental disomy), and cux1_any (any structural alteration). del7q_any was defined as either CNACS-reported arm-level loss involving 7/7q or karyotype patterns of − 7/del(7q).

Co-alteration analyses included both copy-number alterations (loss/gain/UPD) inferred by CNACS and point-mutation data from the IPSS-M source repository. Copy-number co-alterations were assessed for genes on chromosome 7q and selected PRC2 components (*EZH2*, *EED*, *SUZ12*), *TP53*, *TET2*, *ASXL1*, and RAS pathway genes. Point-mutation co-alterations were assessed for recurrently mutated genes (*EZH2*, *TP53*, *KRAS*, *NRAS*, *RUNX1*, *U2AF1*, *SF3B1*, *TET2*, *ASXL1*, *DNMT3A*, *SRSF2*) using Fisher’s exact test with Benjamini-Hochberg false discovery rate (FDR) correction.

### WHO 2022 classification mapping

WHO 2022 classification was retrospectively approximated from WHO 2016 categories, *SF3B1* mutation status, and *TP53* allelic state using the following hierarchy: (1) patients with *TP53* multi-hit were classified as MDS with biallelic *TP53* inactivation (MDS-biTP53); (2) remaining patients were mapped as MDS-EB1 → MDS-IB1, MDS-EB2 → MDS-IB2, MDS-del5q → MDS-5q, MDS-RS subtypes or *SF3B1*-mutated low-blast MDS → MDS-LB-SF3B1, other low-blast MDS → MDS-LB, and MDS-U → MDS-U/NOS. Hypoplastic MDS could not be identified from available data.

### Outcomes

Overall survival (OS) was measured from diagnosis to death from any cause, censoring at last follow-up for survivors. Leukemia-free survival (LFS) was defined from diagnosis to AML transformation or death, whichever occurred first, censoring otherwise.

### Statistical analyses

**Fig. 1:**
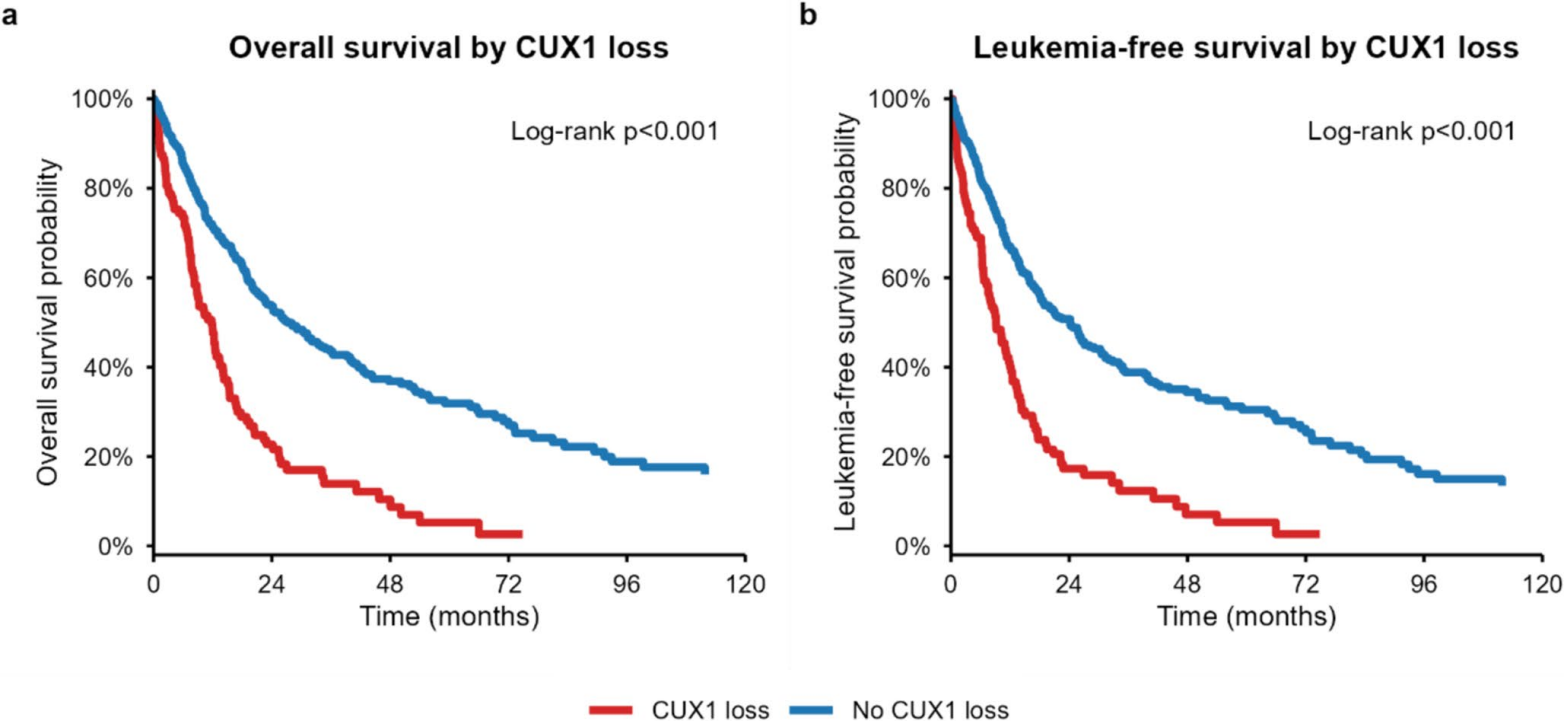
Overall survival and leukemia-free survival by *CUX1* copy-number loss

Kaplan–Meier curves with log-rank tests compared OS and LFS between *CUX1* loss vs. no loss (Fig. 1). Cox proportional hazards models estimated hazard ratios (HRs) for *CUX1* loss: (1) unadjusted; (2) adjusted for age, sex, IPSS-R cytogenetic risk, complex karyotype, and MDS type; and adjusted for age, sex, IPSS-M molecular score, complex karyotype, and MDS type. Proportional hazards assumptions were evaluated using Schoenfeld residuals. Variance inflation factors (VIF) were calculated for to assess collinearity.

To assess whether *CUX1* improves risk prediction beyond IPSS-M, we compared nested Cox models with and without *CUX1* loss, computing likelihood ratio (LR) tests, and Harrell’s concordance (ΔC) via bootstrap resampling (1,000 replicates). Survival within the − 7/del(7q) subgroup was analyzed separately. Fisher’s exact tests produced odds ratios (ORs) and 95% CIs for co-alteration analyses. FDR correction (Benjamini–Hochberg) was applied to co-alteration analyses. All analyses were performed using R, version 4.4.1.

## Results

### Cohort overview

The dataset comprised 3,466 unique patients. The primary analysis cohort contained 501 patients with available CNACS gene-level copy-number calls for *CUX1*, of whom 129 (26%) harbored *CUX1* copy-number loss. Within the primary analysis cohort, we observed 307 OS events and 305 LFS events.

### Baseline characteristics

**Fig. 2:**
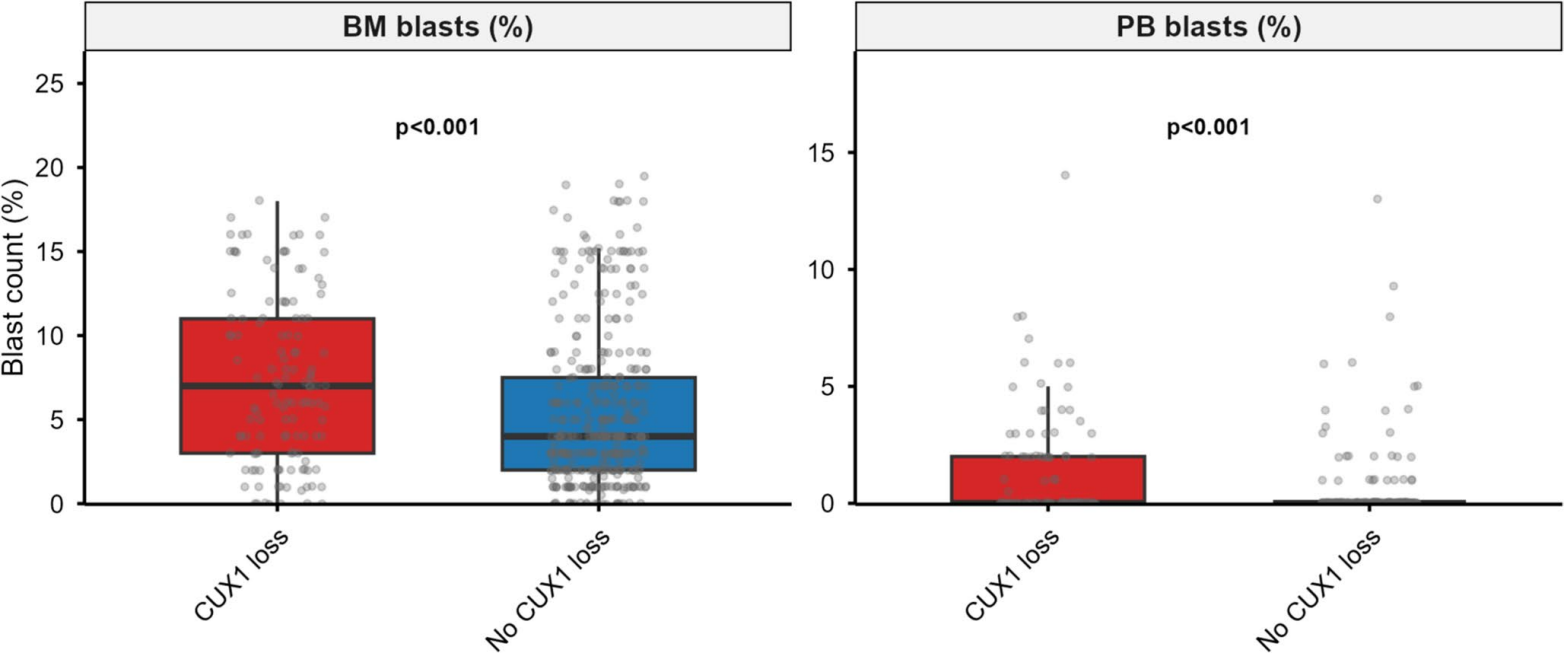
Bone marrow and peripheral blood blast percentage distributions by *CUX1* loss status

Baseline characteristics are shown in Table 1. Patients with *CUX1* loss were slightly younger (mean 66.4 vs. 70.7 years) and more often male (63% vs. 43%). Hemoglobin, WBC, ANC, and platelet counts were all lower in the *CUX1*-loss group. Marrow blasts were higher (median 7% vs. 4%; *P* < 0.001) (Fig. 2). Molecular risk differed strongly: IPSS-R scores averaged 6.9 vs. 4.6 and IPSS-M scores 2.33 vs. 0.79 (both *P* < 0.001; Supplementary Table S2). Complex karyotype was enriched (60% vs. 34%; *P* < 0.001), and − 7/del(7q) was nearly universal(98% vs. 9%; *P* < 0.001). Treatment data were available for 497 patients: 143 (29%) received HMA, 56 (11%) underwent HSCT, and 70 (14%) received lenalidomide. Patients with *CUX1* loss were more likely to receive HMA (44% vs. 24%; *P* < 0.001) and HSCT (21% vs. 8%; *P* < 0.001), but less likely to receive lenalidomide (5% vs. 17%; *P* < 0.001). Under the approximated WHO 2022 classification, *CUX1* loss was strongly underrepresented in MDS-5q (1% vs. 27%; *P* < 0.001) and enriched in MDS-IB2 (25% vs. 15%; *P* = 0.01).

**Table 1:** Baseline characteristics of the *CUX1*-informative cohort

Variable	Overall (*n* = 501)	*CUX1* loss: No (*n* = 372)	*CUX1* loss: Yes (*n* = 129)	*P*-value
n	501	372	129	
Age, years (mean ± SD)	69.6 ± 11.5	70.7 ± 11.4	66.4 ± 11.5	< 0.001
Male, n (%)	242 (48.3)	161 (43.3)	81 (62.8)	< 0.001
Hemoglobin, g/dL (median ^IQR^)	9.01 ^8.04–10.30^	9.10 ^8.20–10.50^	9.00 ^8.00–10.00^	0.149
WBC, G/L (median ^IQR^)	3.20 ^2.40–4.90^	3.60 ^2.60–5.00^	2.95 ^2.00–4.10^	0.001
ANC, G/L (median ^IQR^)	1.51 ^0.87–2.74^	1.71 ^1.00–3.00^	1.00 ^0.56–1.88^	< 0.001
Platelet count, G/L (median ^IQR^)	106.5 ^51.0–202.3^	132.0 ^63.8–236.0^	65.0 ^35.5–103.5^	< 0.001
BM blasts, % (median ^IQR^)	4.00 ^2.00–8.50^	4.00 ^2.00–7.50^	7.00 ^3.00–11.00^	< 0.001
PB blasts, % (median ^IQR^)	0.00 ^0.00–0.00^	0.00 ^0.00–0.00^	0.00 ^0.00–2.00^	< 0.001
IPSS-R score (mean ± SD)	5.19 ± 2.49	4.57 ± 2.45	6.90 ± 1.63	< 0.001
IPSS-M score (mean ± SD)	1.21 ± 1.80	0.79 ± 1.78	2.33 ± 1.30	< 0.001
Complex karyotype, n (%)	201 (40)	125 (34)	77 (60)	< 0.001
−7/del(7q), n (%)	162 (32)	34 (9)	127 (98)	< 0.001
Therapy-related MDS, n (%)	82 (16)	51 (14)	30 (23)	0.033
WHO 2016 class (mean category)	6.65 ± 2.67	6.74 ± 2.74	6.39 ± 2.45	0.191
Treatment (*n* = 497 matched patients)				
HMA, n (%)	143 (29)	87 (24)	56 (44)	< 0.001
Allogeneic HSCT, n (%)	56 (11)	29 (8)	27 (21)	< 0.001
Lenalidomide, n (%)	70 (14)	64 (17)	6 (5)	< 0.001
WHO 2022 Classification (*n* = 497)				
MDS-LB, n (%)	81 (16)	59 (16)	22 (17)	0.781
MDS-LB-SF3B1, n (%)	33 (7)	22 (6)	11 (9)	0.304
MDS-IB1, n (%)	106 (21)	74 (20)	32 (25)	0.258
MDS-IB2, n (%)	87 (17)	55 (15)	32 (25)	0.010
MDS-biTP53, n (%)	68 (14)	44 (12)	24 (19)	0.053
MDS-5q, n (%)	101 (20)	100 (27)	1 (1)	< 0.001
MDS-U/NOS, n (%)	21 (4)	16 (4)	5 (4)	1.000

Continuous variables: median ^IQR^ or mean ± SD. Categorical variables: n (%). P-values: Wilcoxon rank-sum (continuous), Chi-square/Fisher’s exact (categorical)

*BM *bone marrow, *PB* peripheral blood, *WBC* white blood cell count, *ANC* absolute neutrophil count, *IPSS-R* Revised International Prognostic Scoring System, *IPSS-M* Molecular International Prognostic Scoring System, *HMA* hypomethylating agent, *HSCT* allogeneic hematopoietic stem cell transplantation

### Overall and leukemia-free survival

Overall and leukemia-free survival results are shown in Fig. 1; Table 2. *CUX1* loss was associated with markedly inferior survival. Median OS decreased from 27.1 months (no loss) to 11.8 months (loss), with 1-/3-/5-year OS rates of 48%/14%/5% vs. 71%/44%/32% (log-rank *P* < 0.0001). Median LFS likewise declined from 24.2 to 9.2 months (log-rank *P* < 0.0001). Unadjusted HRs were 2.39 (95% CI 1.85–3.08) for OS and 2.30 (1.78–2.98) for LFS. Adjustment for age, sex, IPSS-R category, complex karyotype, and MDS type attenuated but did not eliminate the association (OS HR 1.50 [1.14–1.99]; LFS HR 1.41 [1.07–1.87]).

**Table 2:** Cox proportional hazards models for overall survival and leukemia-free survival

Model	Covariates	OS *n*/events	OS HR (95% CI)	OS *P*	LFS *n*/events	LFS HR (95% CI)	LFS *P*
Unadjusted	None	489/307	2.39 (1.85–3.08)	< 0.001	464/305	2.30 (1.78–2.98)	< 0.001
IPSS-R adjusted	Age, sex, IPSS-R category, complex karyotype, MDS type	446/277	1.50 (1.14–1.99)	0.004	424/272	1.41 (1.07–1.87)	0.016
IPSS-M adjusted	Age, sex, IPSS-M score, complex karyotype, MDS type	411/255	1.27 (0.95–1.70)	0.107	394/254	1.23 (0.92–1.65)	0.154

Hazard ratios (HRs) for *CUX1* loss vs. no *CUX1* loss. IPSS-R: category used as categorical covariate

*IPSS-M* continuous molecular score, *n/events* number of patients with events, *CI* confidence interval, *OS* overall survival, *LFS* leukemia-free survival

### IPSS-M–adjusted models and incremental value

**Fig. 3:**
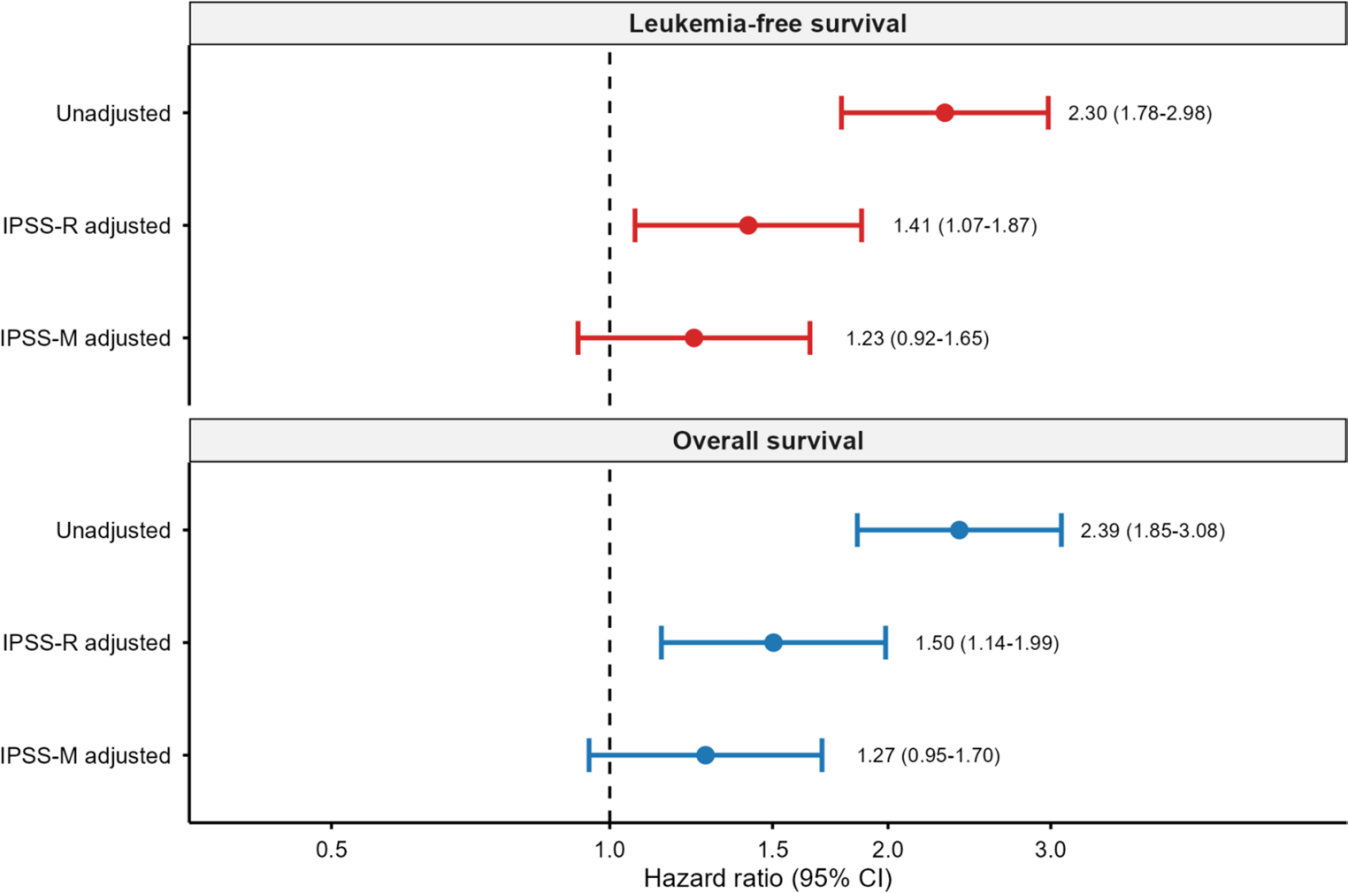
Forest plot of hazard ratios for *CUX1* loss across different Cox models. Forest plot of hazard ratios (95% CI) for CUX1 loss: unadjusted, IPSS-R adjusted, and IPSS-M adjusted models for overall survival (top) and leukemia-free survival (bottom). Dashed line indicates HR=1.

**Fig. 4:**
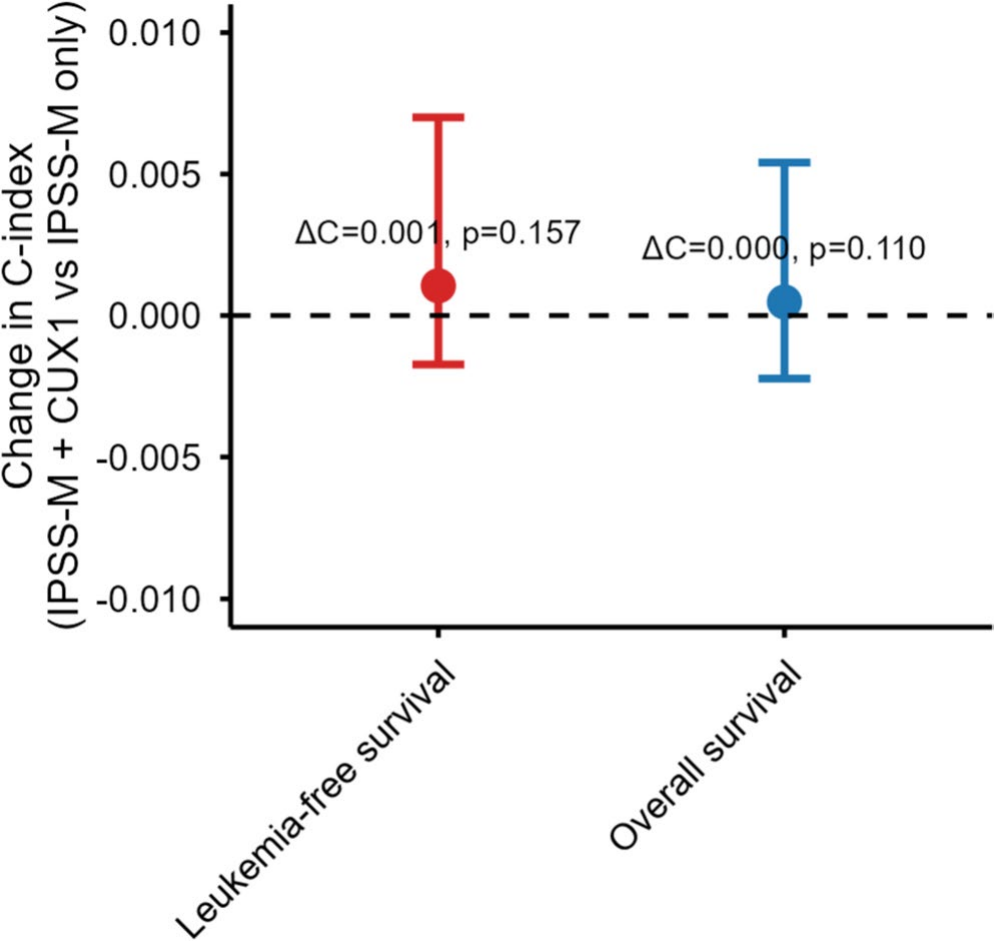
Incremental prognostic value analysis showing change in C-index when adding *CUX1* loss to IPSS-M models

IPSS-M–adjusted models and incremental value analyses are shown in Figs. 3 and 4. After adjusting for age, sex, IPSS-M molecular score, complex karyotype, and MDS type, *CUX1* loss no longer achieved statistical significance (OS HR 1.27 [0.95–1.70], *P* = 0.11; LFS HR 1.23 [0.92–1.65], *P* = 0.15). Adding *CUX1* to IPSS-M models yielded negligible improvements in discrimination (ΔC = + 0.0005 for OS and + 0.0011 for LFS, 95% CIs spanning 0; Supplementary Table S3) and non-significant LR tests (*P* = 0.11 and *P* = 0.16).

### Survival within the − 7/del(7q) subgroup

To evaluate whether the *CUX1* effect was driven by − 7/del(7q), we restricted survival analyses to patients with − 7/del(7q) abnormalities. Among 147 del(7q) patients with available OS data (114 with *CUX1* loss, 33 without), there was no significant difference in OS (median 11.0 vs. 8.8 months; log-rank *P* = 0.41) or LFS (median 9.0 vs. 8.4 months; log-rank *P* = 0.31) (Supplementary Figure S2). In multivariable analysis within the del(7q) subgroup adjusting for IPSS-M score, age, sex, and complex karyotype, *CUX1* loss was non-significant for both OS (HR 1.37, 95% CI 0.86–2.19, *P* = 0.19) and LFS (HR 1.38, 95% CI 0.86–2.22, *P* = 0.19). Notably, *CUX1* loss and − 7/del(7q) were too highly correlated (*r* = 0.84) for inclusion in a single model without collinearity concerns (VIF > 4.5). These findings confirm that the survival impact of *CUX1* loss is attributable to − 7/del(7q) biology.

### Stratified and interaction analyses

Stratified Kaplan–Meier curves are shown in Supplementary Figure S1. *CUX1* loss remained adverse in both Low/Moderate and High/Very-High IPSS-M strata. Exploratory Cox interactions between *CUX1* loss and IPSS-M risk were non-significant (interaction *P* = 0.62), as was the *CUX1*×MDS-type term (*P* = 0.55).

### Co-alteration landscape

Co-alteration results are shown in Table 3 and Supplementary Table S1. *CUX1* loss occurred almost exclusively in the context of − 7/del(7q): 98% of *CUX1* loss cases occurred in patients with − 7/del(7q) versus 9% without (Table 3). PRC2 lesions dominated the gene-level co-alteration spectrum. *EZH2* alterations showed OR 223.8 (95% CI 99.7–549.9; FDR *P* < 0.0001) and PRC2 complex alterations showed OR 60.5 (95% CI 30.7–129.9; FDR *P* < 0.0001). *TP53* alterations displayed moderate enrichment (OR 2.47; FDR *P* < 0.001). In contrast, *TET2*, *ASXL1*, and RAS family alterations yielded ORs near unity (FDR-adjusted *P* ≥ 0.35). Analysis of *TP53* allelic state revealed that *TP53* multi-hit was strongly enriched in *CUX1* loss (50% vs. 27%; OR 2.74, 95% CI 1.81–4.16; *P* < 0.001), whereas *TP53* monoallelic alterations were underrepresented (2% vs. 8%; OR 0.30, 95% CI 0.09–0.99; *P* = 0.03). Chromosome 17 abnormalities were more frequent in *CUX1*-loss patients (37% vs. 19%).

**Table 3:** Co-alteration analysis results for *CUX1* copy-number loss

Gene	CUX1 + Gene+	CUX1 + Gene−	CUX1 − Gene+	CUX1 − Gene−	OR (95% CI)	*P*-value	FDR *P*
*EZH2*	117	12	15	357	223.8 (99.7–549.9)	< 0.001	< 0.001
*PRC2*	117	12	51	321	60.47 (30.7–129.9)	< 0.001	< 0.001
*TP53*	46	83	68	304	2.47 (1.54–3.95)	< 0.001	< 0.001
*EED*	3	126	29	343	0.28 (0.05–0.93)	0.035	0.095
*TET2*	8	121	13	359	1.82 (0.64–4.88)	0.204	0.374
*RAS*	10	119	20	352	1.48 (0.60–3.42)	0.388	0.534
*SUZ12*	2	127	11	361	0.52 (0.06–2.42)	0.530	0.648
*ASXL1*	10	119	29	343	0.99 (0.42–2.18)	1.000	1.000
*TP53* multi-hit	64	63	100	270	2.74 (1.81–4.16)	< 0.001	< 0.001
*TP53* monoallelic	3	124	28	342	0.30 (0.09–0.99)	0.034	0.068

Upper panel: copy-number co-alterations (CNACS data). *PRC2*: *EZH2*, *EED*, or *SUZ12*; *RAS*: *NRAS* or *KRAS*. Lower panel: *TP53* allelic state from the source IPSS-M mutation data (*n* = 497 matched patients). OR, odds ratio; CI, confidence interval; FDR, false discovery rate (Benjamini–Hochberg)

Forest plot of hazard ratios (95% CI) for *CUX1* loss: unadjusted, IPSS-R adjusted, and IPSS-M adjusted models for overall survival (top) and leukemia-free survival (bottom). Dashed line indicates HR = 1

Boxplots of bone marrow (left) and peripheral blood (right) blast percentages: *CUX1* loss (red) vs. no *CUX1* loss (blue). Wilcoxon P-values shown

Change in C-index (ΔC) when adding *CUX1* loss to IPSS-M models for overall survival (blue) and leukemia-free survival (red). Error bars, 95% CI from 1,000 bootstrap resamples. P-values from likelihood ratio tests. Dashed line indicates ΔC = 0

### Point-mutation co-alteration analysis

Point-mutation co-alteration analysis (Supplementary Table S1B) confirmed *EZH2* enrichment (OR 4.57, FDR *P* = 0.004) and revealed additional associations: *KRAS* (OR 10.73, FDR *P* = 0.005), *RUNX1* (OR 2.23, FDR *P* = 0.018), *U2AF1* (OR 2.43, FDR *P* = 0.022), and *TP53* (OR 2.11, FDR *P* = 0.004). *SF3B1* mutations tended toward depletion (OR 0.44, FDR *P* = 0.07). *TET2*, *ASXL1*, *DNMT3A*, and *SRSF2* point mutations showed no significant association, extending the copy-number findings.

## Discussion

Over the past decade, next-generation sequencing testing for patients with MDS has become standard of care, particularly following the IPSS-M scoring system [3]. Prior to the molecular era, reliance on metaphase karyotype alone provided less accurate prognostication.

Patients with *CUX1* loss exhibited features consistent with high-risk MDS biology: higher bone marrow and peripheral blood blast percentages, higher IPSS-R and IPSS-M scores, and increased frequency of complex karyotype, therapy-related disease, and *TP53* multi-hit alterations. These associations align with previous reports linking *CUX1* alterations to aggressive disease phenotypes [12]. The strong association with − 7/del(7q) (present in 98% of *CUX1* loss patients versus 9% without) is biologically plausible given *CUX1*’s location on chromosome 7q22.1 [11]. This near-universal overlap indicates that isolated *CUX1* deletions without broader 7q loss are exceptionally rare (approximately 2% of *CUX1* loss cases), consistent with the contiguous haploinsufficiency model of 7q biology [7, 8].

### Co-alteration landscape: the PRC2 connection

The most striking finding in our co-alteration analysis was the near-universal association between *CUX1* loss and PRC2 complex alterations, particularly *EZH2* (OR 223.8, 95% CI 99.7–549.9). This association is likely explained by the co-localization of both genes on chromosome 7q, where *EZH2* resides at 7q36.1 [7]. When larger deletions encompass both loci, simultaneous loss of *CUX1* and *EZH2* would be expected. The functional significance of this co-deletion warrants further investigation, as both genes play important roles in transcriptional regulation and tumor suppression [11].

In contrast to previous reports suggesting enrichment of *TET2*, *ASXL1*, and RAS family alterations with *CUX1* loss [12], we found no significant associations after FDR correction. These discrepancies may reflect differences in the type of alteration examined. Our study focused on copy-number loss, whereas previous work included point mutations [12]. Notably, *TET2* is predominantly affected by point mutations, which limits assessment of *TET2* co-alterations. using CNA-only data. The point-mutation analysis confirmed: *TET2*, *ASXL1*, *DNMT3A*, and *SRSF2* point mutations showed no significant association with *CUX1* loss.

### TP53 allelic state and multi-hit inactivation

The enrichment of *TP53* multi-hit (but not monoallelic) alterations in *CUX1*-loss patients is notable. Bernard et al. demonstrated that *TP53* allelic state is the critical determinant of clinical behavior in *TP53*-altered MDS, with multi-hit driving adverse outcomes while monoallelic alterations behave similarly to *TP53* wild-type.^16^ The co-occurrence of *TP53* multi-hit with *CUX1* loss likely reflects shared association with complex karyotype and − 7/del(7q) rather than a direct biological interaction.

### Limitations

Several limitations should be acknowledged. First, this was a retrospective analysis of publicly available data, which may introduce selection biases and limit generalizability. Second, although we integrated point mutation data from the source IPSS-M repository, our primary analysis was restricted to copy-number loss; *CUX1* point mutations were rare (7/497, 1.4%) and were not combined with deletions in outcome analyses. *CUX1* downregulation can occur via haploinsufficiency, mutation, or epigenetic mechanisms, with a subset of MDS cases exhibiting reduced expression in the absence of 7q deletions [12]. Our study identifies the prognostic redundancy of physical deletions (which predominantly occur in − 7/del(7q)) but does not rule out *CUX1* expression as an independent driver in chromosomally intact cases. Future studies integrating DNA and RNA sequencing are needed to interrogate this specific subgroup. Additionally, routine detection of − 7/del(7q) lesions relies on cytogenomic workflows recommended by the American College of Medical Genetics and Genomics [17].

Third, we lack functional data to validate therapeutic vulnerabilities. While the strong correlation with PRC2 alterations suggests potential sensitivity to EZH2/PRC2 inhibitors, experimental validation in *CUX1*-haploinsufficient models is required. Treatment data were descriptive; survival models were not adjusted for treatment, as this was not the study aim. The WHO 2022 classification was retrospectively approximated and may not perfectly correspond to prospective classification, particularly for categories requiring bone marrow histological assessment (e.g., hypoplastic MDS).

In summary, our findings confirm that *CUX1* copy-number loss serves as a marker of − 7/del(7q) biology, with strong associations to PRC2/*EZH2* alterations, but does not provide independent prognostic information beyond IPSS-M. While this suggests *CUX1* loss may not warrant inclusion as an independent variable in current prognosis algorithms, validation in independent datasets with uniform treatment annotations is recommended. Ultimately, given the scarcity of actionable mutations in MDS, *CUX1* loss represents an area warranting further investigation, particularly in PRC2-altered MDS.

## Supplementary Information

Below is the link to the electronic supplementary material.

Supplementary Material 1.

## Data Availability

All data used in this study are publicly available from cBioPortal for Cancer Genomics (https://www.cbioportal.org/). Additional molecular data are available from the IPSS-M source repository (https://github.com/papaemmelab/IPSSMstudy).
