# Supplementary material for: Prognostic significance of CUX1 genomic deletion in myelodysplastic neoplasms
Source: Ann Hematol. 2026 Mar 13;105(4):174. doi: 10.1007/s00277-026-06936-y (PMC12987772; doi:10.1007/s00277-026-06936-y)
Supplement: Supplementary file 1 — Supplementary Material 1. [file 277_2026_6936_MOESM1_ESM.docx]

**Supplementary Material**

**Prognostic Significance of CUX1 Genomic Deletion in Myelodysplastic Neoplasms**

Khamis MM, Babic A, Al-Kali A, Alkharabsheh O

## **Supplementary Figure S1. Stratified Kaplan-Meier curves by IPSS-M risk category and MDS type**


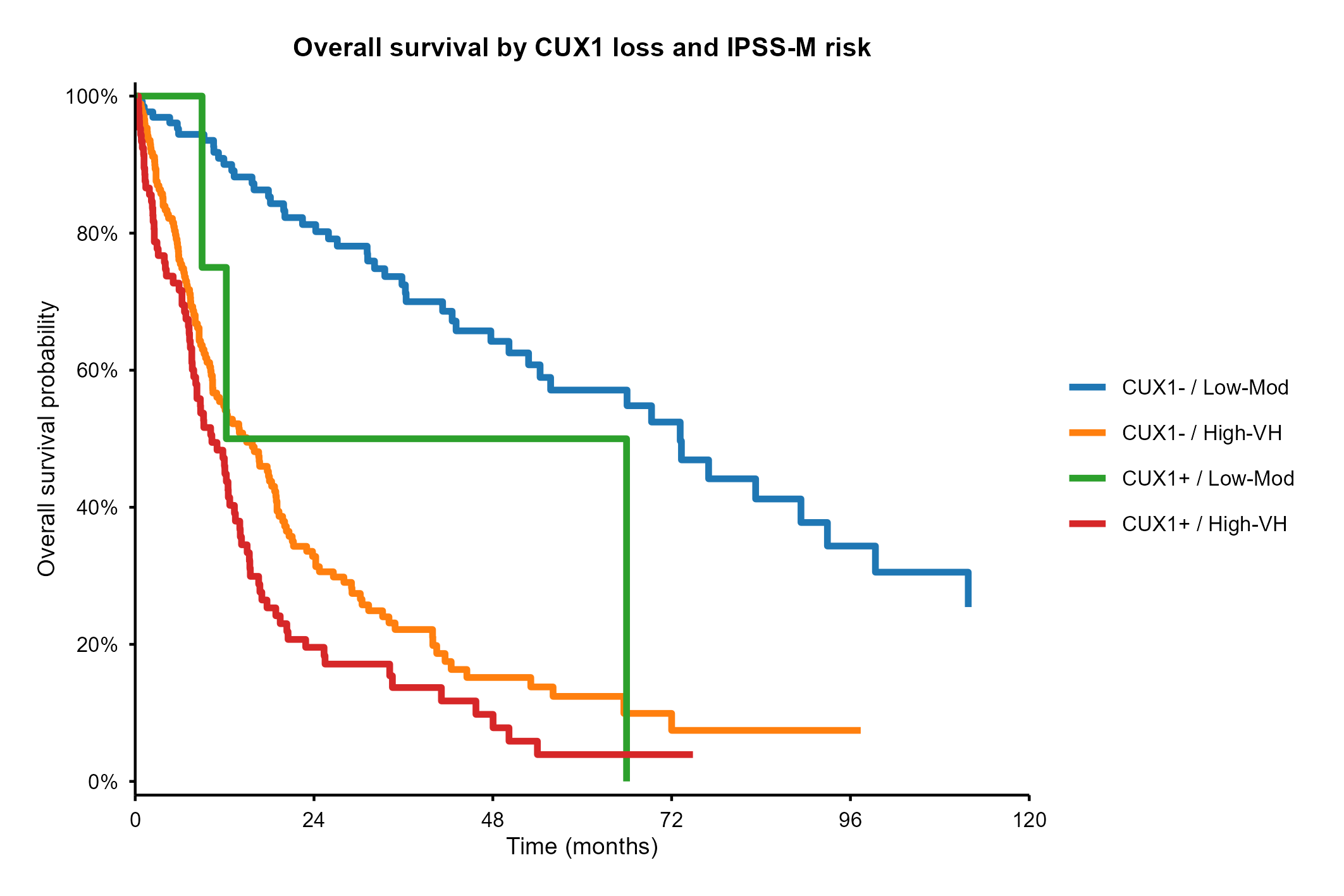


Kaplan-Meier curves for overall survival stratified by *CUX1* loss and IPSS-M risk (low/moderate vs high/very-high). Exploratory analysis. Legend: *CUX1*− / Low-Mod (blue), *CUX1*− / High-VH (orange), *CUX1*+ / Low-Mod (green), *CUX1*+ / High-VH (red).

## **Supplementary Figure S2. Kaplan-Meier curves for overall survival and leukemia-free survival within del(7q) subgroup**


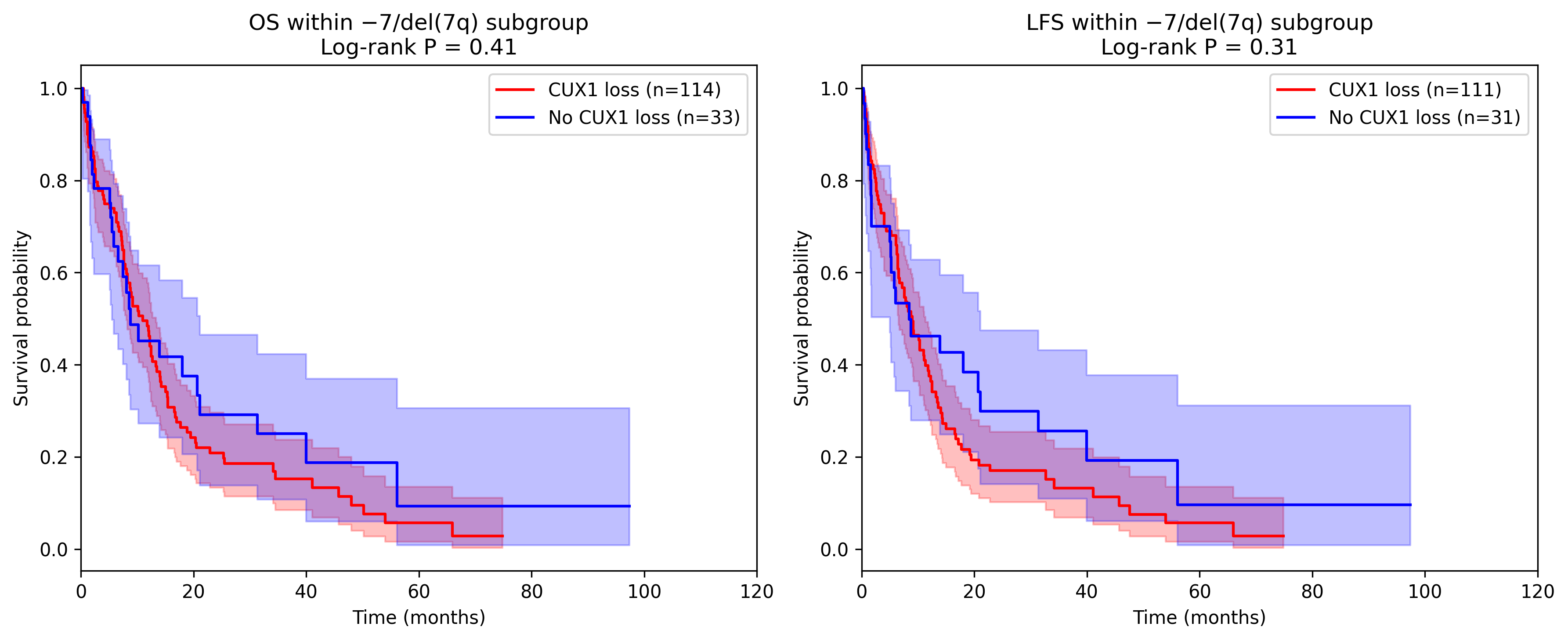


Kaplan-Meier curves for overall survival (left) and leukemia-free survival (right) restricted to patients with −7/del(7q) abnormalities: *CUX1* loss (red, n=114 for OS; n=111 for LFS) vs no *CUX1* loss (blue, n=33 for OS; n=31 for LFS). Log-rank test P-values: OS P = 0.41, LFS P = 0.31. Among del(7q) patients, *CUX1* loss was not significantly associated with survival, confirming that the survival impact of *CUX1* loss in the full cohort is driven by −7/del(7q) biology.

## **Supplementary Table S1. Complete co-alteration analysis results for all tested genes (copy-number alterations)**

| **Gene** | **CUX1+/Gene+** | **CUX1+/Gene−** | **CUX1−/Gene+** | **CUX1−/Gene−** | **OR (95% CI)** | **P-value** | **FDR P** |
| --- | --- | --- | --- | --- | --- | --- | --- |
| EZH2 any | 117 | 12 | 15 | 357 | 223.82 (99.67–549.87) | <0.001 | <0.001 |
| PRC2 any | 117 | 12 | 51 | 321 | 60.47 (30.68–129.89) | <0.001 | <0.001 |
| TP53 any | 46 | 83 | 68 | 304 | 2.47 (1.54–3.95) | <0.001 | <0.001 |
| EED any | 3 | 126 | 29 | 343 | 0.28 (0.05–0.93) | 0.035 | 0.095 |
| TET2 any | 8 | 121 | 13 | 359 | 1.82 (0.64–4.88) | 0.204 | 0.374 |
| RAS any | 10 | 119 | 20 | 352 | 1.48 (0.60–3.42) | 0.388 | 0.534 |
| SUZ12 any | 2 | 127 | 11 | 361 | 0.52 (0.06–2.42) | 0.530 | 0.648 |
| ASXL1 any | 10 | 119 | 29 | 343 | 0.99 (0.42–2.18) | 1.000 | 1.000 |

Complete co-alteration results for all tested copy-number genes. *EED* any: lower frequency (OR < 1); *EZH2* any and PRC2_any: strong enrichment (OR > 50). Priority genes shown in Table 3 of the main manuscript.

## **Supplementary Table S1B. Point mutation co-alteration analysis results**

| **Gene** | **CUX1+/Gene+** | **CUX1+/Gene−** | **CUX1−/Gene+** | **CUX1−/Gene−** | **OR (95% CI)** | **P-value** | **FDR P** |
| --- | --- | --- | --- | --- | --- | --- | --- |
| EZH2 | 17 | 110 | 28 | 342 | 4.574 | <0.001 | 0.0036 |
| TP53 | 27 | 100 | 101 | 269 | 2.111 | <0.001 | 0.0036 |
| KRAS | 8 | 119 | 13 | 357 | 10.733 | <0.001 | 0.0051 |
| RUNX1 | 9 | 118 | 23 | 347 | 2.230 | 0.018 | 0.0182 |
| U2AF1 | 10 | 117 | 25 | 345 | 2.428 | 0.022 | 0.0223 |
| SF3B1 | 2 | 125 | 18 | 352 | 0.440 | 0.069 | 0.0656 |
| TET2 | 7 | 120 | 20 | 350 | 1.068 | 0.977 | 0.9769 |
| ASXL1 | 6 | 121 | 17 | 353 | 1.153 | 0.899 | 0.8994 |
| DNMT3A | 4 | 123 | 14 | 356 | 0.946 | 0.977 | 0.9769 |
| SRSF2 | 3 | 124 | 11 | 359 | 0.868 | 1.000 | 1.0000 |
| NRAS | 2 | 125 | 7 | 363 | 2.244 | 0.328 | 0.3278 |

Point mutation co-alteration results for *CUX1* copy-number loss. FDR-corrected P-values (Benjamini–Hochberg). Significant associations after FDR correction: *EZH2*, *TP53*, *KRAS*, *RUNX1*, *U2AF1* (all FDR P < 0.05). *SF3B1* shows a trend toward depletion (FDR P = 0.0656). *TET2*, *ASXL1*, *DNMT3A*, and *SRSF2* showed no significant association with *CUX1* loss.

## **Supplementary Table S2. Mean IPSS-R and IPSS-M category scores by CUX1 loss status**

| **Variable** | **Overall** | **CUX1 loss: No (n=370)** | **CUX1 loss: Yes (n=127)** | **P-value** |
| --- | --- | --- | --- | --- |
| IPSS-R category (mean±SD) | 3.50 (1.37) | 3.17 (1.37) | 4.43 (0.84) | <0.001 |
| IPSS-M category (mean±SD) | 4.44 (1.76) | 4.03 (1.82) | 5.54 (0.91) | <0.001 |

Mean IPSS-R and IPSS-M category scores (ordinal means ± SD) by *CUX1* loss status. Values represent mean ordinal score on the IPSS-R (1–6) and IPSS-M (1–8) scales. P-values from Mann–Whitney U test.

## **Supplementary Table S3. Detailed incremental value analysis results**

| **Outcome** | **C-index (base)** | **C-index (with CUX1)** | **ΔC-index** | **ΔC CI lower** | **ΔC CI upper** | **LR test χ²** | **LR test P** | **Δ−2logL** |
| --- | --- | --- | --- | --- | --- | --- | --- | --- |
| OS | 0.7645 | 0.7650 | 0.0005 | −0.0022 | 0.0054 | 2.559 | 0.110 | 2.559 |
| LFS | 0.7607 | 0.7617 | 0.0011 | −0.0017 | 0.0070 | 2.000 | 0.157 | 2.000 |

Incremental value analysis: C-index values, ΔC-index (C-index with *CUX1* minus base model), 95% confidence interval, and likelihood ratio (LR) test statistics. CI, confidence interval; LR, likelihood ratio; OS, overall survival; LFS, leukemia-free survival.
